# Subinhibitory antibiotic concentrations promote the horizontal transfer of plasmid-borne resistance genes from *Klebsiellae pneumoniae* to *Escherichia coli*

**DOI:** 10.3389/fmicb.2022.1017092

**Published:** 2022-11-07

**Authors:** Manlin Ding, Zi Ye, Lu Liu, Wei Wang, Qiao Chen, Feiyang Zhang, Ying Wang, Åsa Sjöling, Alberto J. Martín-Rodríguez, Renjing Hu, Wenbi Chen, Yingshun Zhou

**Affiliations:** ^1^Department of Pathogenic Biology, School of Basic Medicine, Southwest Medical University, Luzhou, China; ^2^Department of Clinical Laboratory, The Affiliated Traditional Chinese Medicine Hospital of Southwest Medical University, Luzhou, China; ^3^Department of Microbiology, Tumor and Cell Biology, Karolinska Institutet, Stockholm, Sweden; ^4^Department of Laboratory Medicine, The Affiliated Wuxi No. 2 People’s Hospital of Nanjing Medical University, Wuxi, China

**Keywords:** antibiotic, sub-MIC, plasmid, conjugation frequency, T4SS, metabolome

## Abstract

Horizontal gene transfer plays an important role in the spread of antibiotic resistance, in which plasmid-mediated conjugation transfer is the most important mechanism. While sub-minimal inhibitory concentrations (sub-MIC) of antibiotics could promote conjugation frequency, the mechanism by which sub-MIC levels of antibiotics affect conjugation frequency is not clear. Here, we used *Klebsiella pneumoniae* SW1780 carrying the multi-drug resistance plasmid pSW1780-KPC as the donor strain, to investigate the effects of sub-MICs of meropenem (MEM), ciprofloxacin (CIP), cefotaxime (CTX), and amikacin (AK) on conjugational transfer of pSW1780-KPC from SW1780 to *Escherichia coli* J53. Our results showed that the transfer frequencies increased significantly by treating SW1780 strain with sub-MIC levels of MEM, CIP, CTX and AK. Transfer frequencies at sub-MIC conditions in a *Galleria mellonella* were significantly higher than *in vitro*. To investigate gene expression and metabolic effects, RT-qPCR and LC–MS-based metabolome sequencing were performed. Transcript levels of T4SS genes *virB1*, *virB2*, *virB4*, *virB8*, and conjugation-related genes *traB*, *traK*, *traE*, and *traL* were significantly upregulated by exposure to sub-MICs of MEM, CIP, CTX, and AK. Metabolome sequencing revealed nine differentially regulated metabolites. Our findings are an early warning for a wide assessment of the roles of sub-MIC levels of antibiotics in the spread of antibiotic resistance.

## Introduction

In 1928, Griffith first described horizontal gene transfer (HGT; Griffith, 1928) and discovered a way for bacteria to acquire foreign DNA. HGT occurs *via* three different mechanisms: transformation, transduction, and conjugation. Of these, conjugation is most frequent (Lopatkin et al., 2016; von Wintersdorff et al., 2016; Jesús et al., 2018) and is largely driven by plasmids (Rensen et al., 2005; Prensky et al., 2021), which are transferred from one bacterial cell to another by conjugative pili (Hu et al., 2019). Plasmids commonly carry antibiotic resistance genes or virulence genes, which are therefore transferred from donor bacteria to recipient bacteria, thereby increasing bacterial fitness and representing a powerful tool in bacterial evolution and adaptability (Thomas and Nielsen, 2005; Smalla et al., 2015).

In Gram-negative bacteria, the type 4 secretory system (T4SS) is a multifunctional complex that is widely distributed and closely related to conjugation (Cabezón et al., 2015; Grohmann et al., 2018). The horizontal transfer of ARGs is one of the main reasons for the global dissemination of antibiotic resistance in the environment, a problem that is becoming increasingly serious (Gogarten et al., 2002; Liu et al., 2020; Zhang et al., 2022). In the clinics, administration of antibiotics requires the consideration of pharmacokinetic parameters and therefore prescribed doses are higher than the minimum inhibitory concentration (MIC). However, the concentration of antibiotics at the site of infection is often lower than the MIC, which is defined as sub-minimal concentration (sub-MIC; Odenholt, 2001; Romero et al., 2011). Antibiotics at sub-MIC levels are unable to inhibit bacterial proliferation; in contrast, they can induce profound morphological and physiological changes in the microbes (Gardner, 1940; Lorian, 1975; Wojnicz et al., 2007; Abdelazizahmed and Elbannatarek, 2019), including increased acquisition of antibiotic resistance genes by HGT (Baharoglu et al., 2013; Zhang et al., 2018). As early as 1986, evidence that β-lactam antibiotics increase the frequency of plasmid transfer in *Staphylococcus aureus* by phage-mediated conjugation was provided (Barr et al., 1986). More recently, sub-MIC of CIP or LEV were shown to significantly increase the conjugation frequency of a model plasmid from *Escherichia coli* to *Pseudomonas aeruginosa* (Shun-Mei et al., 2017). A recent study (Wen et al., 2021) found that 1 mg/L of doxycycline could down-regulate the fitness cost of competition between drug-resistant bacteria and sensitive bacteria, among which the related down-regulated biomarkers were pyruvate and pilocarpine. In general, the impact of sub-MIC levels of β-lactam and aminoglycoside antibiotics on conjugation transfer frequency remain largely uncharacterized, and the metabolic effects of sub-MIC doses of these antibiotics on bacterial populations are largely unknown. It is the purpose of this study to shed light on these questions.

Thus, in this work, we investigated whether sub-MIC antibiotic levels are able to promote conjugative transfer of ARGs carried by a plasmid across bacterial genera. We used *in vitro* and *in vivo* (*Galleria mellonella* larvae) model systems, to study inter-genera plasmid transfer from *Klebsiella pneumoniae* SW1780 to the model recipient strain *E*. *coli* J53. Our results demonstrated that sub-MICs of meropenem, ciprofloxacin, cefotaxime and amikacin could significantly enhance conjugative transfer frequency both *in vitro* and in the *G*. *mellonella* model systems.

## Materials and methods

### Strain and medium

In 2018, *K*. *pneumoniae* SW1780 was isolated from the sputum sample of ICU patient in Henan, China. *E. coli* J53 is a model recipient strain resistant to sodium azide, and was retrieved from our laboratory collection.

### Whole-genome sequencing

Genomic DNA of *K*. *pneumoniae* SW1780 was extracted using the Wizard® Genomic DNA Purification Kit (Promega, United States) according to manufacturer’s protocol. PacBio RS II (~10 K) libraries and Illumina PE (400 bp) libraries were sequenced using a PacBio RS II platform and an Illumina Hiseq platform, respectively. *De novo* assembly was performed from sequencing reads. RAST v.2.0^1^ was used to complete bacterial genome annotations. The virulence factor database VFDB^2^ was used to predict virulence factors. ResFinder v.4.1 and PlasmidFinder v.2.1, available from the Center for Genomic Epidemiology^3^, were used to predict resistance genes to identify plasmids, respectively. The oriT finder software^4^ was used to identify putative origin of transfers in DNA sequences of bacterial mobile genetic elements.

### Antimicrobial susceptibility testing

*In vitro* antimicrobial susceptibility tests of meropenem, cefotaxime, ciprofloxacin, and amikacin, were determined using the broth microdilution method following recommendations of the Clinical and Laboratory Standards Institute (CLSI).^5^

### Determination of antibiotic subinhibitory concentrations

Antibiotic sub-MICs were selected by creating a growth curve. *K*. *pneumoniae* SW1780 was grown in the presence of 0 × MIC, 1/2 × MIC, 1/4 × MIC, 1/8 × MIC, 1/16 × MIC, 1/ 32 × MIC, 1/64 × MIC, 1/128 × MIC, 1/256 × MIC, 1/512 × MIC, 1/1024 × MIC, 1/2048 × MIC of meropenem (YuanYeBio-Technology, Shanghai, China), ciprofloxacin (Sango Biotech, Shanghai, China), cefotaxime (Sango Biotech, Shanghai, China) and amikacin (Sango Biotech, Shanghai, China). Samples were collected at one-hour intervals and the optical density was read at 595 nm (OD595) using a microplate reader (Bio-Rad, United States). The logarithmic growth rate of the antibiotic stress group and the non-antibiotic group was calculated by applying the logarithmic growth rate formula: U = [ln(Nt) -ln(N0)]/(t-t0), where Nt is the logarithmic growth late OD_595_ value, N0 is the logarithmic growth early OD_595_ value, and t-t0 is the logarithmic period time. One-way ANOVA was used to determine statistical differences. The highest antibiotic concentration that did not affect the growth of strain SW1780 was used as sub-MIC.

### *In vitro* conjugation assay under sub-MIC pressure

*Klebsiellae pneumoniae* SW1780 was exposed to the presence or absence of sub-MIC antibiotics for 6 h. Phosphate Buffered Saline (PBS) was used to wash the cells and remove residual antibiotics. Then, donor *K*. *pneumoniae* SW1780 and recipient *E*. *coli* J53 were incubated in LB medium for 13 h at 37°C. In order to screen transconjugants, 100 μL of the suspensions were then plated on the selective LB plates containing meropenem (0.3 μg/mL) and sodium azide (100 μg/mL) and incubated for 24 at 37°C. Conjugation frequencies were calculated by dividing the number of transconjugants (the number of colonies on the selective plate which contain meropenem and sodium azide) by the number of *E*. *coli* J53 recipients (the number of colonies on the selective plate only contain sodium azide). Three separate experiments were performed and plates with CFUs between 30 and 300 colonies were included in this assay.

The colony PCR method was used to verify the identity of single colonies grown on the selective medium. Thus, the plasmid-brone carbapenemase gene *bla*KPC (F: CGGGATCCATGTCACTGTATCGCCGTC, R: CGGAATTCTTACTGCCCGTTAACGCC) and the 16S rRNA gene (F: AGAGTTTGATYM TGGCTCAG, R: GYTACCTTGTTACGACTT) were amplified and the PCR products were submitted to Sanger sequencing to identify the bacterial species. The antimicrobial susceptibility of the transconjugants was detected by the microbroth dilution method.

### *In vivo* conjugation assay under sub-MIC pressure

*Galleria mellonella* (Tianjin Huiyude Biotechnology) weighing 200-250 mg and maintained in the dark at 8–10°C were employed in all assays. To determine the optimal concentration of SW1780 and J53 inocula to be employed in assays with *G*. *mellonella*, we prepared cell suspensions of both strains in PBS (pH = 7.4) ranging from 1 × 10^5^ cfu/mL to 1 × 10^8^ cfu/mL. The prepared bacterial solutions or a mock solution composed by PBS only, were injected (10 μL) into the body cavity of *G*. *mellonella* larvae using a microsyringe through the right-hind foot. The PBS control group was injected with 10 μL PBS buffer. The blank control group received no treatment. The five groups were incubated in Petri dishes at 37°C for 72 h. The survival status of the six groups was recorded at 24, 48, and 72 h post-inoculation. Each experiment was repeated three times.

*In vivo* conjugation: the method for treating SW1780 with sub-MIC antibiotics is the same as *in vitro*. After the treatment, the concentration of the bacterial suspension of SW1780 and J53 was adjusted to the corresponding optimal concentration in *G*. *mellonella*, and injected into the right-hind foot of the larvae. Following incubation in the dark at 37°C for 24 h, larvae homogenates were suspended and diluted with PBS and used to inoculate on meropenem (0.3 μg/mL) and sodium azide (100 μg/mL) selection plates as well as selection plates containing only sodium azide (100 μg/mL). After 48 h of incubation, the transconjugants were counted and the conjugation frequency was calculated.

### Real-time fluorescence quantitative PCR

Total bacterial RNA was extracted from 3 mL of *K*. *pneumoniae* SW1780 cultures after 6 h of sub-MIC antibiotics treatment using the Total RNA Isolation kit (Sangon Biotech Co., Ltd., Shanghai), according to the recommendations of the manufacturer. This 6 h-treated strain was named SW1786. Real-time PCR was carried out using the *TransStart*® Green qPCR SuperMix (TransGen Biotech, Beijing, China) in a real-time PCR instrument (CFX 96, Bio-Rad, Hercules, CA, United States). The primers used in the present study are shown in Supplementary Table S2. The real-time PCR mixtures consisted of 10 μL of 2X *TransStart*® Green qPCR SuperMix, 0.4 μL of each primer (10 μM final concentrations), 1 μL of cDNA template, and 8.2 μL of nuclease-free H2O. The thermocycling profile for the amplifications was 94°C for 30 s, followed by 40 cycles of 94°C for 5 s, 55°C for 15 s, and a melting curve analysis at 72°C for 10 s. Each experiment was conducted at least by triplicate. The housekeeping gene 16 s rRNA was used as an internal reference gene. The 2^−△△CT^ method was used to calculate changes in transcription levels of conjugation-associated genes (*traB*, *traK*, *traE*, *traL*) and T4SS genes (*virB1*, *virB2*, *virB4*, and *virB8*) in cells treated with sub-MIC concentrations of meropenem, ciprofloxacin, cefotaxime and amikacin.

### Metabolome

Cell pellets (control group: no treatment; experimental group: 0.125 μg/mL meropenem treated for 6 h) were collected by centrifugation of bacterial cultures in 50 mL centrifuge tubes after gentle washing with 10 mL precooled PBS solution, three times. After centrifugation, the supernatant was discarded and the pellet stored at-80°C. Each group was set to five replicates. The sample extracts were analyzed using a Liquid Chromatography Mass Spectrometry (LC–MS) system, and the data were analyzed on the Majorbio Cloud Platform.

### Statistical analyses

All statistical analyses were performed with GraphPad Prism 8.0. The statistical significance of the differences in conjugation frequencies were examined by One-way ANOVA. The log-rank test for multiple comparisons were used to calculate the differences in survival. *p* values <0.05 were considered statistically significant.

## Results

### Analysis of the genomic content of *Klebsiellae pneumoniae* SW1780 reveals the existence of a multidrug resistance plasmid

The complete whole-genome sequencing of *K*. *pneumoniae* SW1780 revealed a genome composed by a chromosome with a size of 5,492,736 bp and a GC content of 57.37%, as well as two circular plasmids, namely plasmid A (pSW1780-KPC), with a size of 125,619 bp and a GC content of 54.17%, and plasmid B with a size of 10,060 bp and a GC content of 55.06%.

Drug-resistance genes, and their chromosomal or plasmid locations are presented in Table 1. Three drug resistance genes were located on the chromosome, whereas six were in plasmid pSW1780-KPC. The DNA sequences were uploaded to the online database VFDB, and a total of 25 virulence factors were predicted.

**Table 1:** *Klebsiella pneumoniae* SW1780 antibiotic resistance genes.

Location	Resistance genes
Chromosome	*oqxA*, *bla*SHV-182, *fosA*
	*bla*CTX-M-65, *bla*KPC-2, *bla*TEM-1B
Plasmid A (pSW1780-KPC)	*fosA3*, *catA2*, *rmtB*

**Figure 1:**
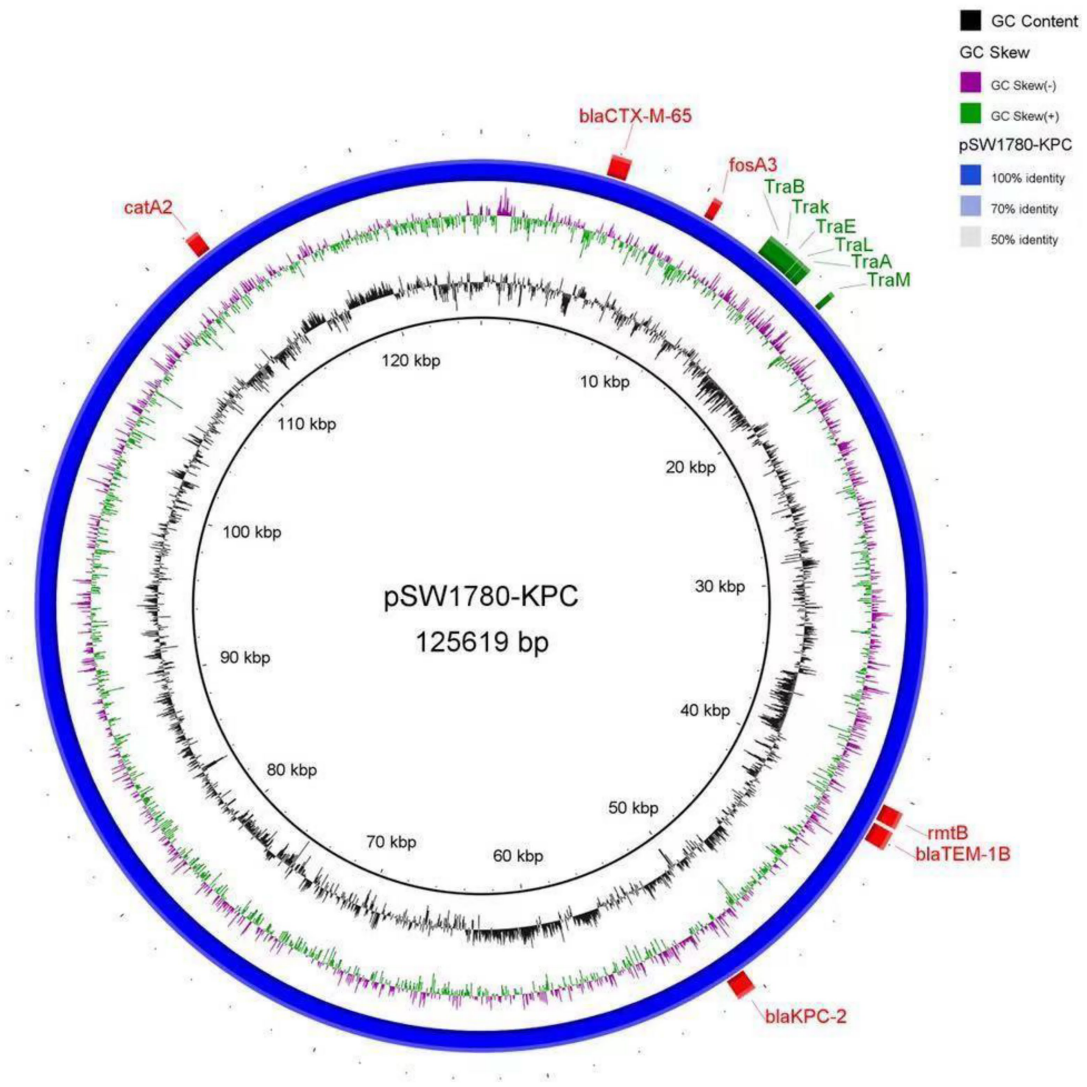
DNA sequence diagram of plasmid pSW1780-KPC. The first circle represents GC content. The second circle indicates the GC skew. The blue circle represents the whole sequence of the plasmid. Externally to the blue circle, red boxes represent antibiotic resistance genes, and green boxes represent conjugation-related genes.

Further analysis of the plasmid showed that pSW1780-KPC is an IncFII type plasmid. The transfer initiation region (oriT) was identified at positions 17,413 to 17,498 by oriT Finder, including the conjugation-related genes *traB*, *traK*, *traE*, *traL*, *traA* and *traM* (Figure 1).

### Antibiotics sub-MICs increase conjugation frequency between *Klebsiellae pneumoniae* SW1780 and *Escherichia coli* J53

To investigate the effect of sub-MICs on plasmid conjugal transfer, the MIC values of meropenem, cefotaxime, ciprofloxacin and amikacin against strain SW1780 were determined by the broth microdilution method, including *E*. *coli* ATCC 25922 as a reference (Table 2). Next, for the selection of appropriate sub-MICs, we analyzed SW1780 growth curves and calculated logarithmic growth rates in the presence of serial two-fold dilutions of meropenem ranging from 1/2 × MIC to 1/1024 × MIC. Thus, growth of SW1780 as measured as total cell density, as well as logarithmic growth rate, were significantly different from the control group under 1/2 × MIC meropenem pressure (*p* < 0.05). Therefore 1/4 × MIC meropenem was used as the maximum sub-MIC concentration (Supplementary Figures S1, S2). In the same way, 1/2 × MIC ciprofloxacin, 1/4 × MIC cefotaxime and 1/2 × MIC amikacin were used as the maximum subinhibitory concentrations of these antibiotics (Supplementary Figures S1, S2). Specific subinhibitory concentrations of antibiotics are shown in Supplementary Table S1.

**Table 2:** Minimum inhibition concentrations of four antibiotics.

		MIC (μg/mL)	
Antibiotics	*K*. *pneumoniae* SW1780	*E*. *coli* ATCC 25,922	*E*. *coli* J53
Meropenem	128	0.0625	0.25
Cefotaxime	512	0.03125	1
Ciprofloxacin	6,4	0.0125	0.25
Amikacin	2,048	2	4

**Figure 2:**
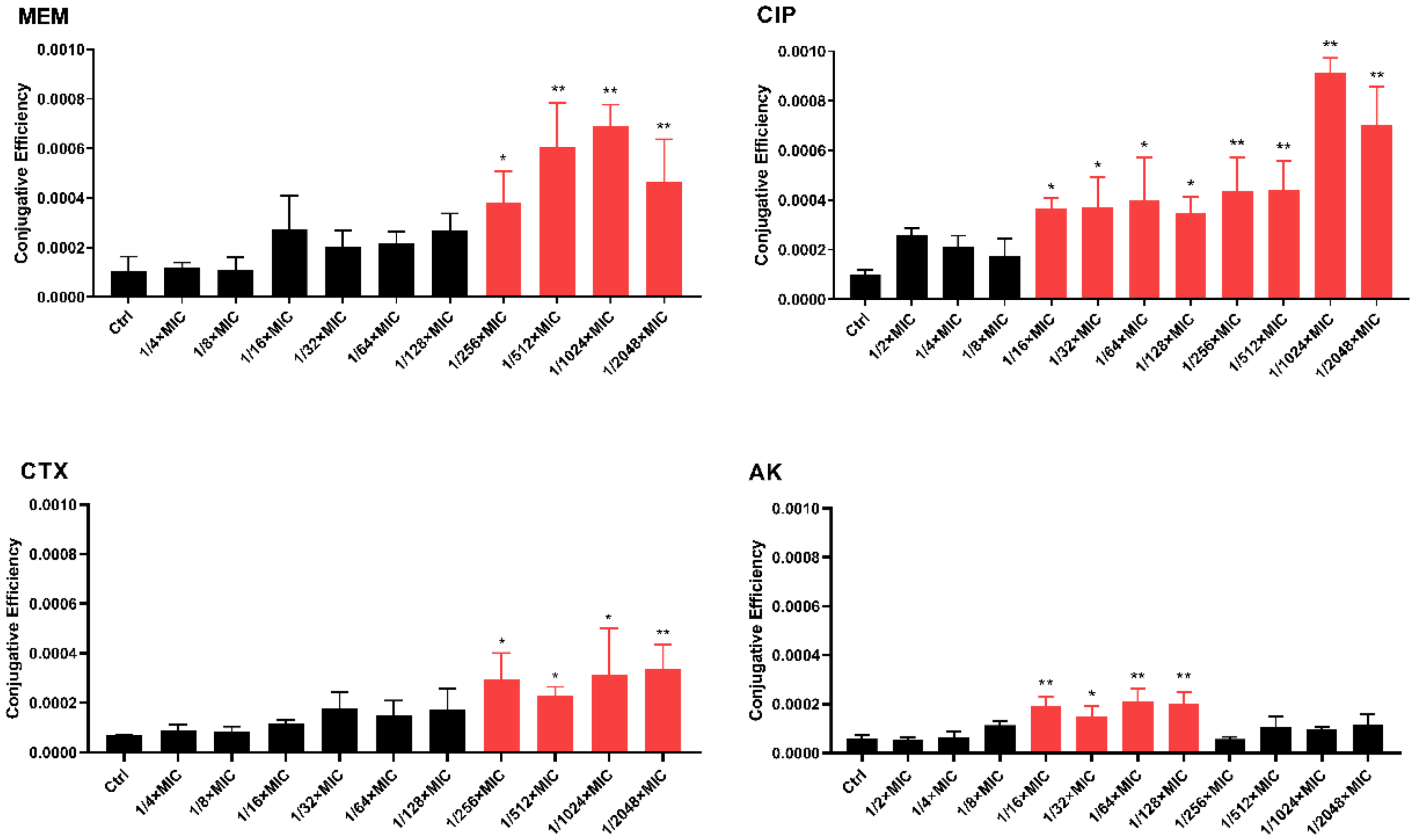
*In vitro* conjugation frequency under expose to antibiotic sub-MICs. The error bar shows the standard deviation. The stars indicate statistical significance at different levels: *P < 0.05, **P < 0.01.

We next tested the effect of antibiotic sub-MICs on conjugation frequency between *K*. *pneumoniae* SW1780 and *E*. *coli* J53 in the presence of sub-MICs of meropenem (MEM), ciprofloxacin (CIP), cefotaxime (CTX) and amikacin (AK). Figure 2 compiles the results of these of these experiments. Four sub-MICs (1/256 × MIC, 1/512 × MIC, 1/1024 × MIC, 1/2048 × MIC) of meropenem increased conjugation frequency by 2.258, 3.597, 4.084 and 2.760 times, respectively. Eight sub-MICs (1/16 × MIC, 1/32 × MIC, 1/64 × MIC, 1/128 × MIC, 1/256 × MIC, 1/512 × MIC, 1/1024 × MIC, 1/2048 × MIC) of ciprofloxacin increased conjugation frequency by 3.575, 3.634, 3.918, 3.424, 4.279, 4.315, 8.960 and 6.873 times, respectively. Four sub-MICs (1/256 × MIC, 1/512× MIC, 1/1024 × MIC, 1/2048 × MIC) of cefotaxime increased conjugation frequency by 4.210, 3.296, 4.451 and 4.784 times, respectively. Finally, four sub-MICs (1/16 × MIC, 1/32 × MIC, 1/64 × MIC, 1/128 × MIC) of amikacin increased conjugation frequency by 3.126, 2.426, 3.483 and 3.291times, respectively. Of note and in contrast to all other antibiotics, amikacin concentrations higher than 1/128 × MIC did not elicit significant effects on plasmid conjugal transfer compared to the control. Collectively, these results indicate that sub-MICs of β-lactam and aminoglycoside antibiotics significantly increase conjugation frequency *in vitro*.

### Antibiotics sub-MICs increase conjugation frequency *Galleria mellonella* larvla model

**Figure 3:**
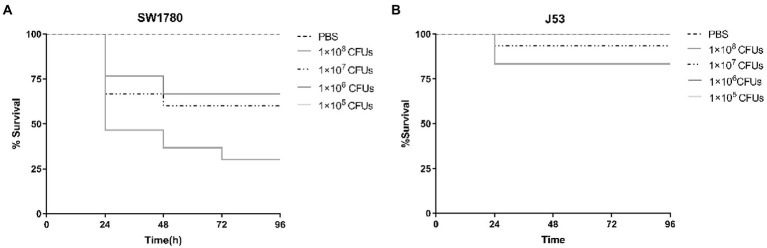
Survival of larval larvae infected with strains *K*. *pneumoniae* SW1780 **(A)** and *E*. *coli* J53 **(B)**.

To investigate the effect of antibiotic sub-MICs *in vivo*, we employed a *G*. *mellonella* larval model. To this end, we first characterized the lethal effects of strains SW1780 and J53 on *G*. *mellonella* larvae using 10-fold growing bacterial inocula from 10^5^ to 10^8^ CFU/mL (Figure 3). Larval death rates at 24 h post-inoculation were proportional to the density of the inocula for both strains, and were notably more pronounced for SW1780. Thus, for SW1780 cell suspensions containing 1 × 10^8^, 1 × 10^7^, 1 × 10^6^, or 1 × 10^5^ CFUs, the 24-h survival rates of *G*. *mellonella* larvae were 46.67, 66.67, 76.67, and 100%, respectively, whereas for J53 survival rates were 83.33, 93.33, 100, and 100%, respectively. Thus, the selected cell densities of SW1780 and J53 for the *in vivo* conjugation assays were 1 × 10^5^ CFUs and 1 × 10^6^ CFUs, respectively.

**Figure 4:**
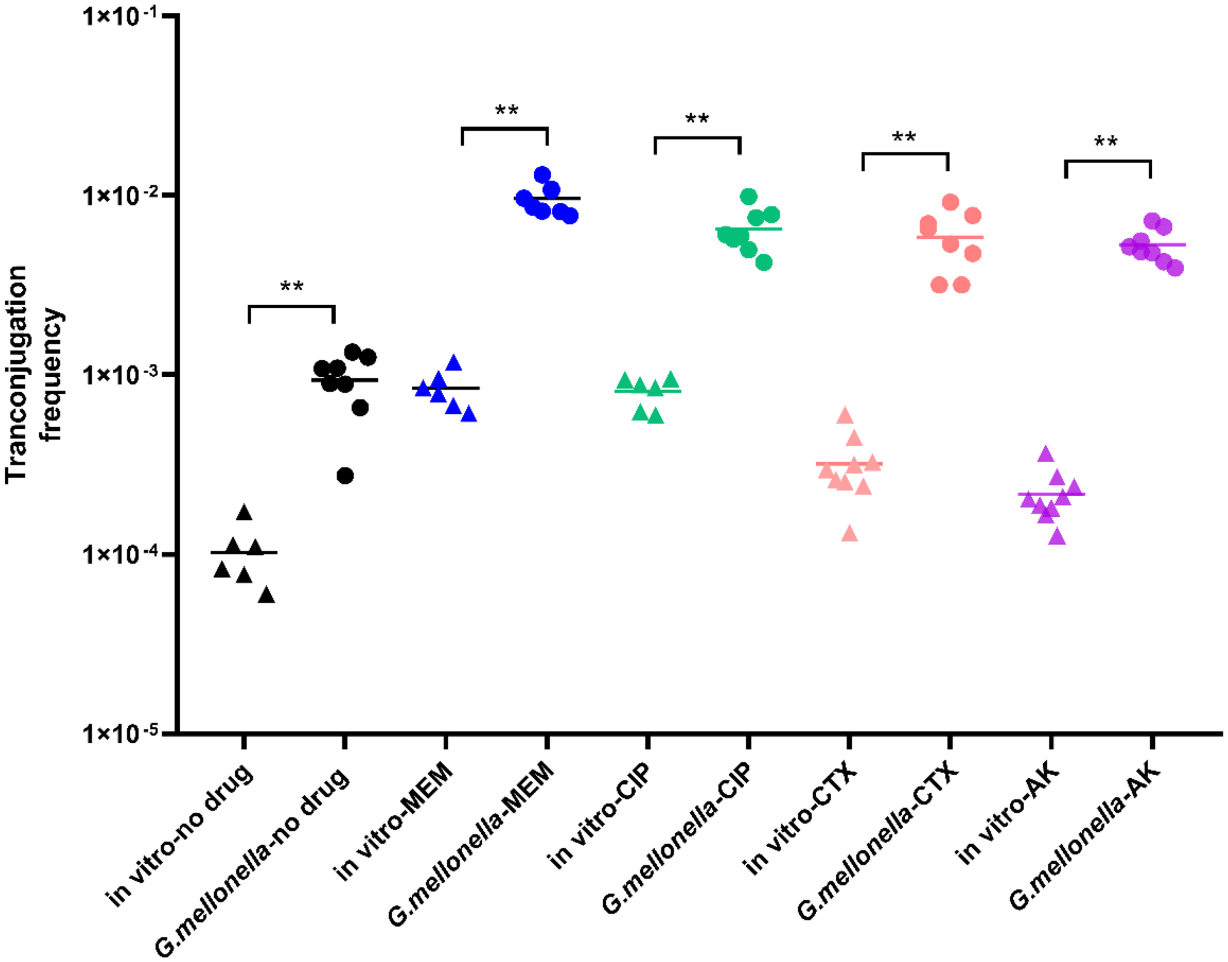
Conjugation frequency in *G*. *mellonella*. For comparison, *in vitro* transconjugation frequencies are shown. The stars indicate statistical significance at different levels: *P < 0.05, **P < 0.01.

As it can be seen from Figure 4, in the absence of antibiotics, the conjugation frequency *in vivo* (9.353 × 10^−4^ ± 0.000345) was significantly higher than that *in vitro* (1.02 × 10^−4^ ± 0.000014), representing a 9-fold increase (*p* < 0.01). In *G*. *mellonella*, the 1/1024 MIC meropenem treatment group (9.488 × 10^−3^ ± 0.0055), 1/1024 MIC ciprofloxacin treatment group (5.623 × 10^−3^ ± 0.00259), 1/2048 MIC cefotaxime treatment group (5.857 × 10^–3^ ± 0.002137) and 1/64 MIC amikacin treatment groups (5.312 × 10^−3^ ± 0.00114) showed significantly higher conjugation frequencies (5.6-10-fold) than the control group (*p* < 0.01). The conjugation frequency of the meropenem treatment group was 9.565 times higher *in vivo* than *in vitro*. Likewise, the conjugation frequencies of the ciprofloxacin, cefotaxime and amikacin treatment groups were 8.021, 18.361 and 24.593 times higher than the respective *in vitro* treatment groups.

### Expression of conjugation genes was up-regulated under exposure to sub-MIC levels of antibiotics

**Figure 5:**
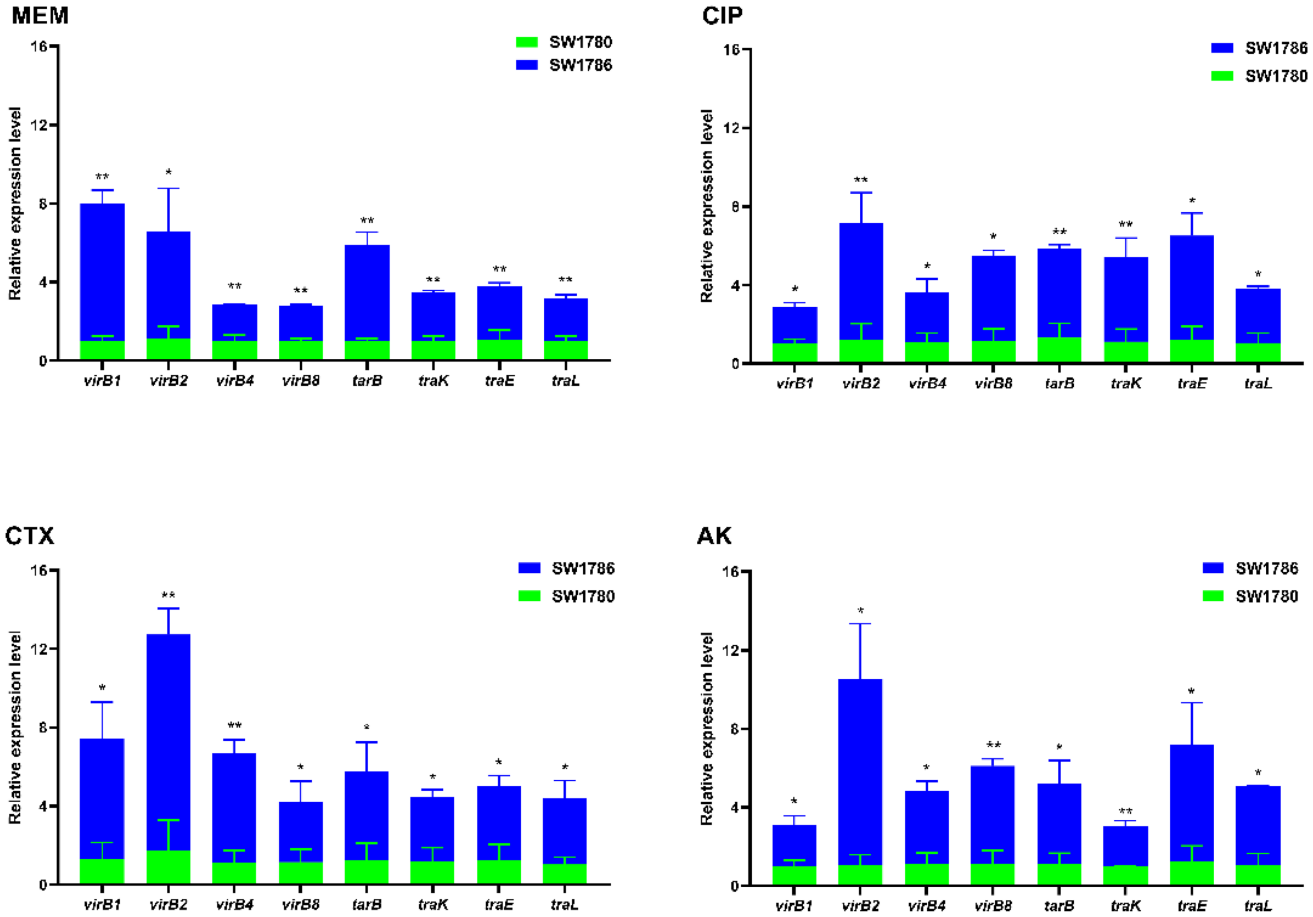
Transcript levels of T4SS and conjugation genes after treatment with sub-MIC antibiotics compared to untreated controls. The error bar shows the standard deviation. The stars indicate statistical significance at different levels: *P < 0.05, **P < 0.01. SW1786 refers to strain SW1780 after 6-h exposure to different antibiotics.

**Figure 6:**
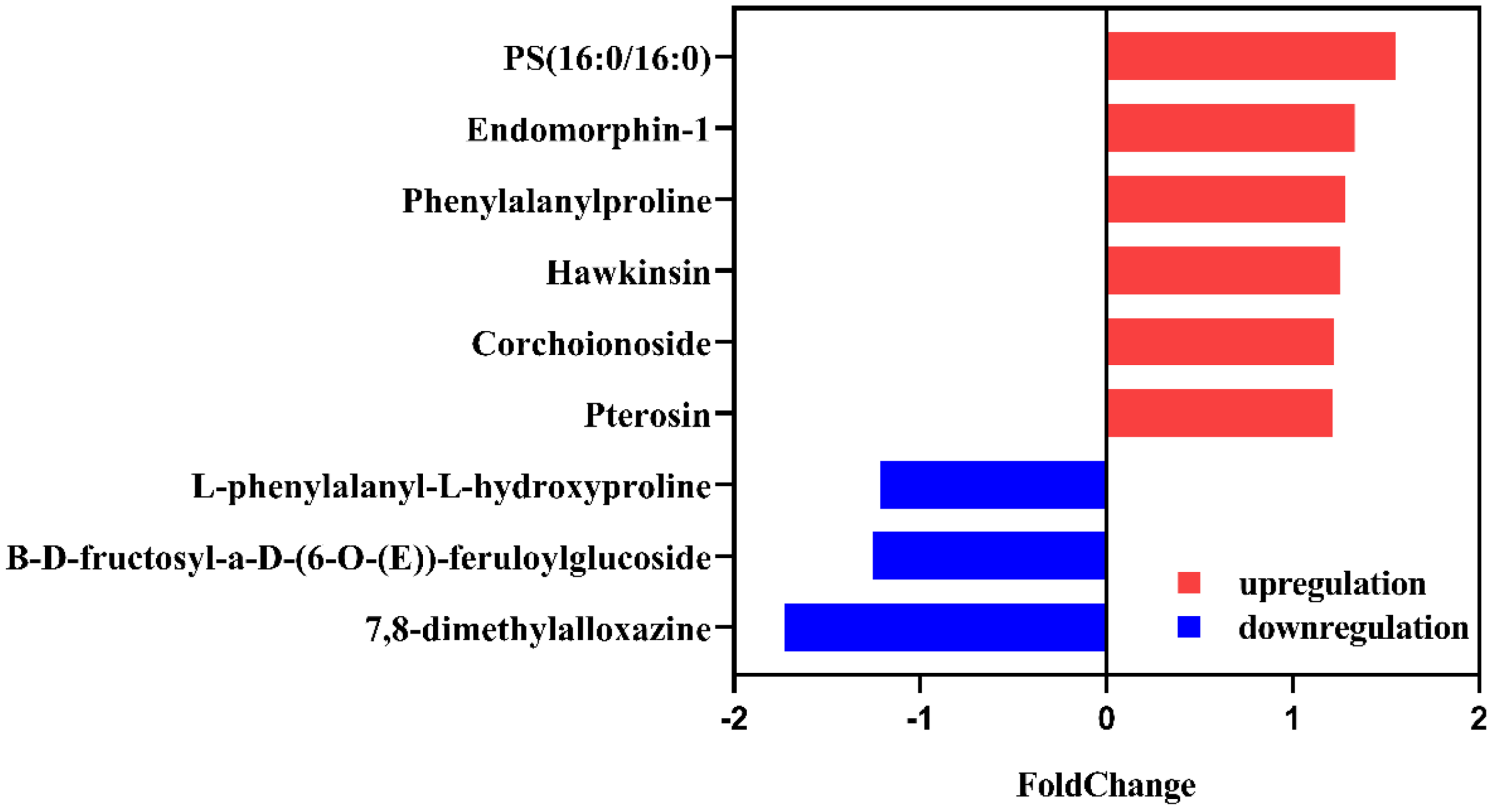
Differentially produced metabolites in meropenem-free cultures of *K*. *pneumoiae* SW1780 and under sub-MIC levels of meropenem (SW1786 vs. SW1780).

As it can be seen from Figure 5, after 6 h treatment with 1/1024 × MIC (0.125 μg/mL) MEM, 1/1024 × MIC (0.0625 μg/mL) CIP, 1/2048 × MIC (0.25 μg/mL) CTX, 1/64 × MIC (128 μg/mL) AK, the expressions of T4SS genes *virB1*, *virB2*, *virB4*, *virB8*, and the conjugation genes *traB*, *traK*, *traE* and *traL* were up-regulated (*p* < 0.05). Among them, under MEM treatment, *virB1* gene upregulation was the most significant, which was 6.97 times, and under the CIP, CTX and AK treatments, *virB2* was upregulated by 5.923 times, 10.997 times and 9.46 times, respectively. Note that in Figure 6, strain SW1780 exposed to antibiotics for 6 h was renamed “SW1786.”

### Differential metabolites in meropenem-free and subinhibitory concentrations of meropenem may cause a change in the conjugation frequency

*Klebsiella pneumoniae* SW1780 differentially produced an array of metabolites in the absence of meropenem compared to in the presence of sub-MIC levels of this antibiotic. According to the screening criteria of differentially produced metabolites (fold change ≤0.833 or ≥ 1.2, *p* < 0.05), 60 and 57 differential metabolites were identified in the positive and negative mode, respectively (Supplementary Figure S3). Details on the identity of these metabolites and their differential production are shown in Figure 6 and Table 3.

**Table 3:** Detailed table of differential metabolites in meropenem-free and subinhibitory concentrations of meropenem.

No.	Metabolite	M/Z^c^	Mode	Formula	Retention time	Log2FC (SW1786/SW1780)	*P*_value	Variatio trend (SW1786/SW1780)
1	7,8-dimethylalloxazine	523.1237	Pos^a^	C_12_H_10_N_4_O_2_	4.1390	−0.788	0.004556	↓
2	Phenylalanylproline	245.1280	Pos	C_14_H_18_N_2_O_3_	3.8740	1.2814	0.001219	↑
3	B-D-fructosyl-a-D-(6-O-(E))-feruloylglucoside	437.1485	Pos	C_21_H_28_O_12_	3.6116	0.7985	0.00295	↓
4	Pterosin J	235.0862	Pos	C_14_H_17_ClO_2_	3.8935	1.2153	0.001045	↑
5	PS (16:0/16:0)	756.4870	Neg^b^	C_38_H_74_NO_10_P	11.2093	1.5517	0.04325	↑
6	Endomorphin-1	631.2632	Neg	C_34_H_38_N_6_O_5_	8.6796	1.3323	0.002773	↑
7	Hawkinsin	312.0505	Neg	C_11_H_17_NO_6_S	1.4909	1.2577	0.02385	↑
8	L-phenylalanyl-L-hydroxyproline	313.0940	Neg	C_14_H_18_N_2_O_4_	3.694	0.8231	0.01152	↓
9	Corchoionoside B	445.1719	Neg	C_19_H_28_O_9_	1.2181	1.2188	0.0148	↑

a Positive ion.

b Negative ion.

c Mass-to-charge ratio.

## Discussion

With the rapid development of sequencing technologies, whole genome sequencing has become a major tool to study the genetic composition and the function of key genes in bacteria. In this study, the whole genome of the multi-drug resistant *K*. *pneumoniae* strain SW1780 was sequenced using the second and third generation molecular sequencing techniques. The strain carried 9 drug-resistant genes, and 6 of them (*bla*TEM-1B, *fosA3*, *bla*KPC-2, *catA2*, *bla*CTX-M-65, *rmtB*) were located on a plasmid named pSW1780-KPC. The resistance genes carried in the plasmid are likely the main reason for the multiple drug resistance of strain SW1780. Through bioinformatic analysis, it was found that plasmid pSW1780-KPC has a complete conjugation transfer system which allows for its conjugal transfer.

In this study, sub-MIC levels of amikacin, meropenem, ciprofloxacin and cefotaxime increased the frequency of plasmid conjugation *in vitro*. At present, many studies have shown that sub-MIC levels of antibiotics can promote the horizontal transfer of drug-resistant genes. Thus, sub-MICs of ciprofloxacin and levofloxacin significantly promoted the conjugation frequency of *E*. *coli* RP4 plasmid into *P. aeruginosa* in a dose-dependent manner after 8 h treatment (Shun-Mei et al., 2017). Recent studies have shown that tetracycline (1/150 × MIC) inc reases the conjugation transfer frequency by about four times compared to frequencies recorded in the absence of the antibiotic, suggesting that sub-MIC of tetracycline can promote horizontal transfer of antibiotic resistance genes (Kim et al., 2014).

*G*. *mellonella* represents a convenient, inexpensive and high-throughput pathogenic bacterial infection model, with reported similarities with the natural immune system of mammals and an ability to thrive at 37°C, thus representing a suitable host for the study of human pathogens (Cook and McArthur, 2013; Selvam et al., 2015). The lack of ethical concerns around its use in experimentation facilitates the implementation of this model in different laboratories. Therefore, the influence of donor bacteria and recipient bacteria on the conjugation frequency *in vivo* was explored by constructing a model of pathogen infection and a conjugation model using *G*. *mellonella* larvae. The conjugation frequency of *bla*OXA-48 was 8.7 × 10^−7^ *in vitro* and 1.3 × 10^−4^ *in vivo*, which was 149.425 times higher than that *in vitro* (Göttig et al., 2015). In a mouse model, conjugal transfer rates of *bla*OXA-48 in the intestinal tract of experimental mice was found to be 2.9 × 10^−5^, 33.333 times higher than that *in vitro* (Göttig et al., 2015).

In our study, after 6 h treatment with 1/1024 × MIC (0.125 μg/mL) meropenem, 1/1024 × MIC (0.0625 μg/mL) ciprofloxacin, 1/2048 × MIC (0.25 μg/mL) cefotaxime and 1/64 × MIC (0.0625 μg/mL) amikacin, the expression of the T4SS genes *virB1*, *virB2*, *virB4*, *virB8*, as well as the expression of the conjugation genes *traB*, *traK*, *traE*, *traL*, was up-regulated. In Gram-negative bacteria, T4SS is a multifunctional complex that is widely present and closely related to conjugation and transfer systems. Most of the T4SS pili are tubular structures composed of VirB2 and VirB5 (Fronzes et al., 2009), which can be used as a channel for DNA transport from donor to recipient bacteria. The increased expression of conjugation-related and T4SS-related genes could be one of the mechanisms by which the sub-MIC of meropenem promoted conjugation transfer in *K*. *pneumoniae* SW1780.

The synthesis of beta-lactamases, particularly the *K*. *pneumoniae* carbapenemase (KPC), which has been extensively reported in *K*. *pneumoniae* isolates, is one of the most prevalent resistance mechanisms among Enterobacterales (Romanelli et al., 2021). Our bacterial metabolomic data can offer insight about how bacterial metabolism changes in the presence of subinhibitory concentrations of antibiotics, which has been addressed by few studies at present. Metabolomic analysis revealed that sub-MIC of doxycycline alleviates fitness costs of plasmid-bearing resistant *E*. *coli* strains (Wen et al., 2021). This phenomenon could be related to the down-regulation of the two biomarkers, pyruvate and pilocarpine (Wen et al., 2021). In this study, 9 heteroproxies were selected, including 7,8-dimethylalloxazine, phenylalanylproline, B-D-fructosyl-a-D-(6-O-(E))-feruloylglucoside, pterosin J, PS (16,0/16:0), endomorphin 1, hawkinsin, L-Phenylalanyl-L-hydroxyproline, corchoionoside. Of these, 7, 8-dimethylalloxazine is a photodegradation product of riboflavin (van Galen et al., 2020). The downregulation of this metabolite in the presence of meropenem at sub-MIC levels suggests that the decomposition of riboflavin is reduced. Riboflavin is the precursor of the cofactor flavin adenine dinucleotide (FAD), involved in its reduced form FADH_2_ as an electron donor in the electron transport chain. This result could potentially point to alterations in bacterial respiration induced by antibiotic sub-MIC, which requires further investigation. The role of bacterial energetics on biofilm formation and antibiotic resistance is currently in the spotlight (Martín-Rodríguez, 2022).

In summary, the results showed that the sub-MIC of MEM, CIP, CTX and AK significantly increased conjugation frequency. We found that sub-MIC of MEM, CIP, CTX and AK could simultaneously affect T4SS genes expression of the donor and the expression of conjugation-related genes in the transferable plasmid.

## Data availability statement

These Whole Genome Shotgunprojects for strainSW1780 has been deposited in DDBJ/EMBL/GenBank under the sequence accession number GCA_018140905.1.

## Author contributions

MD: data curation and writing-original draft. ZY and LL: methodology. WW and QC: formal acquisition. FZ and YW: data curation. ÅS and AM-R: writing-review and editing. RH, WC, and YZ: funding acquisition. All authors contributed to the article and approved the submitted version.

## Funding

This research was funded by the Sichuan Province Science and Technology Project (2020YJ0338), Natural Science Foundation of Luzhou (2021-NYF-20), Southwest Medical University Project (21YYJC0529 and 2021ZKQN130). AJM-R acknowledges funding from Karolinska Institutet (Junior Investigator Award Ref. 2022–00021).

## Conflict of interest

The authors declare that the research was conducted in the absence of any commercial or financial relationships that could be construed as a potential conflict of interest.

## Publisher’s note

All claims expressed in this article are solely those of the authors and do not necessarily represent those of their affiliated organizations, or those of the publisher, the editors and the reviewers. Any product that may be evaluated in this article, or claim that may be made by its manufacturer, is not guaranteed or endorsed by the publisher.

## Footnotes

^1^ http://rast.nmpdr.org/rast.cgi/

^2^ http://www.mgc.ac.cn/VFs/

^3^ http://cge.cbs.dtu.dk/services/

^4^ https://tool-mml.sjtu.edu.cn/oriTfinder/oriTfinder.html

^5^ https://clsi.org/

## References

[ref1] Abdelazizahmed S. Elbannatarek E. (2019). Exposure to sublethal concentrations of Benzalkonium chloride induces antimicrobial resistance and cellular changes in *Klebsiellae pneumoniae* clinical isolates. Microb. Drug Resist. 25, 631–638. doi: 10.1089/mdr.2018.0235, PMID: 30614757

[ref2] Baharoglu Z. Garriss G. Mazel D. (2013). Multiple pathways of genome plasticity leading to development of antibiotic resistance. Antibiotics 2, 288–315. doi: 10.3390/antibiotics2020288 27029305 PMC4790341

[ref3] Barr V. Barr K. Millar M. R. Lacey R. W. (1986). Beta-lactam antibiotics increase the frequency of plasmid transfer in *Staphylococcus aureus*. J. Antimicrob. Chemother. 17, 409–413. doi: 10.1093/jac/17.4.409, PMID: 3710955

[ref4] Cabezón E. Ripoll-Rozada J. Peña A. de la Cruz F. Arechaga I. (2015). Towards an integrated model of bacterial conjugation. FEMS Microbiol. Rev. 39, 81–95. doi: 10.1111/1574-6976.12085, PMID: 25154632

[ref5] Cook S. M. McArthur J. D. (2013). Developing *Galleria mellonella* as a model host for human pathogens. Virulence 4, 350–353. doi: 10.4161/viru.25240, PMID: 23799664 PMC3714126

[ref6] Fronzes R. Christie P. J. Waksman G. (2009). The structural biology of type IV secretion systems. Nat. Rev. Microbiol. 7, 703–714. doi: 10.1038/nrmicro2218, PMID: 19756009 PMC3869563

[ref7] Gardner A. D. (1940). Morphological effects of penicillin on bacteria. Nature 146, 837–838. doi: 10.1038/146837b0

[ref8] Gogarten J. P. Doolittle W. F. Lawrence J. G. (2002). Prokaryotic evolution in light of gene transfer. Mol. Biol. Evol. 19, 2226–2238. doi: 10.1093/oxfordjournals.molbev.a004046 12446813

[ref9] Göttig S. Gruber T. M. Stecher B. Wichelhaus T. A. Kempf V. A. (2015). *In vivo* horizontal gene transfer of the carbapenemase OXA-48 during a nosocomial outbreak. Clin. Inf. Dis. 60, 1808–1815. doi: 10.1093/cid/civ191, PMID: 25759432

[ref10] Griffith F. (1928). The significance of pneumococcal types. J. Hyg. 27, 113–159. doi: 10.1017/S0022172400031879, PMID: 20474956 PMC2167760

[ref11] Grohmann E. Christie P. J. Waksman G. Backert S. (2018). Type IV secretion in gram-negative and gram-positive bacteria. Mol. Microbiol. 107, 455–471. doi: 10.1111/mmi.13896, PMID: 29235173 PMC5796862

[ref12] Hu B. Khara P. Christie P. J. (2019). Structural bases for F plasmid conjugation and F pilus biogenesis in *Escherichia coli*. Proc. Natl. Acad. Sci. U. S. A. 116, 14222–14227. doi: 10.1073/pnas.1904428116, PMID: 31239340 PMC6628675

[ref13] Jesús B. Jerónimo R.-B. Ivan M. (2018). Antibiotic-induced genetic variation: how it arises and how it can BePrevented. Annu. Rev. Microbiol. 72, 209–230. doi: 10.1146/annurev-micro-090817-062139 30200850

[ref14] Kim S. Yun Z. Ha U. H. Lee S. Park H. Kwon E. E.. (2014). Transfer of antibiotic resistance plasmids in pure and activated sludge cultures in the presence of environmentally representative micro-contaminant concentrations. Sci. Total Environ. 468, 813–820. doi: 10.1016/j.scitotenv.2013.08.100, PMID: 24076502

[ref15] Liu Y. Tong Z. Shi J. Jia Y. Yang K. Wang Z. (2020). Correlation between exogenous compounds and the horizontal transfer of plasmid-borne antibiotic resistance genes. Microorganisms. 8. doi: 10.3390/microorganisms8081211, PMID: 32784449 PMC7463591

[ref16] Lopatkin A. J. Huang S. Smith R. P. Srimani J. K. Sysoeva T. A. Bewick S.. (2016). Antibiotics as a selective driver for conjugation dynamics. Nat. Microbiol. 1:16044. doi: 10.1038/nmicrobiol.2016.44, PMID: 27572835 PMC5010019

[ref17] Lorian V. (1975). Some effects of subinhibitory concentrations of antibiotics on bacteria. Bull. N. Y. Acad. Med. 51, 1046–1055. PMID: 1058727 PMC1749612

[ref18] Martín-Rodríguez A. J. (2022). Respiration-induced biofilm formation as a driver for bacterial niche colonization. Trends Microbiol. doi: 10.1016/j.tim.2022.08.007. Epub ahead of print., PMID: 36075785

[ref19] Odenholt I. (2001). Pharmacodynamic effects of subinhibitory antibiotic concentrations. Int. J. Antimicrob. Agents 17, 1–8. doi: 10.1016/S0924-8579(00)00243-0, PMID: 11137642

[ref20] Prensky H. Gomez-Simmonds A. Uhlemann A. C. Lopatkin A. J. (2021). Conjugation dynamics depend on both the plasmid acquisition cost and the fitness cost. Mol. Syst. Biol. 17:e9913. doi: 10.15252/msb.20209913, PMID: 33646643 PMC7919528

[ref21] Rensen S. R. J. Mark B. Hansen L. H. Niels K. Stefan W. (2005). Studying plasmid horizontal transfer in situ: a critical review. Nat. Rev. Microbiol. 3, 700–710. doi: 10.1038/nrmicro1232 16138098

[ref22] Romanelli F. Stolfa S. Morea A. Ronga L. Prete R. D. Chironna M.. (2021). Meropenem/vaborbactam activity *in vitro*: a new option for *Klebsiella pneumoniae* carbapenemase (KPC)-producing *Klebsiella pneumoniae* treatment. Future Microbiol. 16, 1261–1266. doi: 10.2217/fmb-2021-0007 34674551

[ref23] Romero D. Traxler M. F. López D. Kolter R. (2011). Antibiotics as signal molecules. Chem. Rev. 111, 5492–5505. doi: 10.1021/cr2000509, PMID: 21786783 PMC3173521

[ref24] Selvam R. M. Nithya R. Devi P. N. Shree R. S. B. Nila M. V. Demonte N. L.. (2015). Exoproteome of aspergillus flavus corneal isolates and saprophytes: identification of proteoforms of an oversecreted alkaline protease. J. Proteome 115, 23–35. doi: 10.1016/j.jprot.2014.11.017, PMID: 25497218

[ref25] Shun-Mei E. Zeng J. M. Yuan H. Lu Y. Chen C. (2017). Sub-inhibitory concentrations of fluoroquinolones increase conjugation frequency. Microb. Pathog. 114, 57–62. doi: 10.1016/j.micpath.2017.11.036 29174700

[ref26] Smalla K. Jechalke S. Top E. M. (2015). Plasmid detection, characterization, and ecology. Microbiol Spectr. 3:3.1.17. doi: 10.1128/microbiolspec.PLAS-0038-2014, PMID: 26104560 PMC4480600

[ref27] Thomas C. M. Nielsen K. M. (2005). Mechanisms of, and barriers to, horizontal gene transfer between bacteria. Nat. Rev. Microbiol. 3, 711–721. doi: 10.1038/nrmicro1234 16138099

[ref28] van Galen C. Barnard D. T. Stanley R. J. (2020). Stark spectroscopy of Lumichrome: A possible candidate for stand-off detection of bacterial quorum sensing. J. Phys. Chem. B 124, 11835–11842. doi: 10.1021/acs.jpcb.0c09498, PMID: 33325706 PMC8714027

[ref29] von Wintersdorff C. J. Penders J. van Niekerk J. M. Mills N. D. Majumder S. van Alphen L. B.. (2016). Dissemination of antimicrobial resistance in microbial ecosystems through horizontal gene transfer. Front. Microbiol. 7:173. doi: 10.3389/fmicb.2016.00173 26925045 PMC4759269

[ref30] Wen X. Cao J. Mi J. Huang J. Liang J. Wang Y.. (2021). Metabonomics reveals an alleviation of fitness cost in resistant *E*. *coli* competing against susceptible *E*. *coli* at sub-MIC doxycycline. J. Hazard. Mater. 405:124215. doi: 10.1016/j.jhazmat.2020.124215, PMID: 33109407

[ref31] Wojnicz D. Kłak M. Adamski R. Jankowski S. (2007). Influence of subinhibitory concentrations of amikacin and ciprofloxacin on morphology and adherence ability of uropathogenic strains. Folia Microbiol. 52, 429–436. doi: 10.1007/BF02932099, PMID: 18062193

[ref32] Zhang B. Hu R. Liang Q. Liang S. Li Q. Bai J.. (2022). Comparison of two distinct subpopulations of *Klebsiella pneumoniae* ST16 co-occurring in a single patient. Microbiol Spectr. 10:e0262421. doi: 10.1128/spectrum.02624-21, PMID: 35467408 PMC9241866

[ref33] Zhang Y. Ma Q. Su B. Chen R. Lin J. Lin Z.. (2018). A study on the role that quorum sensing play in antibiotic-resistant plasmid conjugative transfer in *Escherichia coli*. Ecotoxicology 27, 209–216. doi: 10.1007/s10646-017-1886-0, PMID: 29350317

