# Supplementary material for: Subinhibitory antibiotic concentrations promote the horizontal transfer of plasmid-borne resistance genes from Klebsiellae pneumoniae to Escherichia coli
Source: Front Microbiol. 2022 Nov 7;13:1017092. doi: 10.3389/fmicb.2022.1017092 (PMC9678054; doi:10.3389/fmicb.2022.1017092)
Supplement: Supplementary file 1 [file Table_1.docx]

| Antibiotic | sub-MIC (μg/mL) | | | | | | | | | | | |
| --- | --- | --- | --- | --- | --- | --- | --- | --- | --- | --- | --- | --- |
|  | MIC | 1/2MIC | 1/4MIC | 1/8MIC | 1/16MIC | 1/32MIC | 1/64MIC | 1/128MIC | 1/256MIC | 1/512MIC | 1/1024MIC | 1/2048MIC |
| Meropenem | 128 | 64 | 32 | 16 | 8 | 4 | 2 | 1 | 0.5 | 0.25 | 0.125 | 0.0625 |
| Cefotaxime | 512 | 256 | 128 | 64 | 32 | 16 | 8 | 4 | 2 | 1 | 0.5 | 0.25 |
| Ciprofloxacin | 64 | 32 | 16 | 8 | 4 | 2 | 1 | 0.5 | 0.25 | 0.125 | 0.0625 | 0.03125 |
| Amikacin | 2048 | 1024 | 512 | 256 | 128 | 64 | 32 | 16 | 8 | 4 | 2 | 1 |

# Table S1. Antibiotic sub-MICs
