# Supplementary material for: Subinhibitory antibiotic concentrations promote the horizontal transfer of plasmid-borne resistance genes from Klebsiellae pneumoniae to Escherichia coli
Source: Front Microbiol. 2022 Nov 7;13:1017092. doi: 10.3389/fmicb.2022.1017092 (PMC9678054; doi:10.3389/fmicb.2022.1017092)
Supplement: Supplementary file 2 [file Table_2.docx]

| primer | sequence（5’-3’） | product size（bp） | origin |
| --- | --- | --- | --- |
| *virB1*-F | CGCTGAGTTGTTATTATTC | 106 | This study |
| *virB1*-R | TCACTGGCAGGGGCGGTTT |  |  |
| *virB2*-F | TAAGACCCTGTGGAAAGGC | 112 | This study |
| *virB2*-R | TCATCAGCAGAGCGCCGAT |  |  |
| *virB4*-F | TGCTCAAGGCAATGAAACG | 109 | This study |
| *virB4*-R | AGAACAGGAGTGCGACGGA |  |  |
| *virB8*-F | TCGCCGTGGTCGTGATGTT | 113 | This study |
| *virB8*-R | TTGCGGTCAGGCTGGTTTT |  |  |
| *tarB*-F | GTGCAGCCTCCGCGATTGGT | 109 | This study |
| *tarB*-R | TGCCTGCTGACGCACCTTG |  |  |
| *tarK*-F | GCAGGCTGGTATCAGGTCC | 115 | This study |
| *tarK*-R | TCAGGGCAGACAGCGTTT |  |  |
| *tarE*-F | GGGTGAATAACTACCGTCTT | 102 | This study |
| *tarE*-R | ACGGAGGGCAATAAATGA |  |  |
| *tarL*-F | CGGGAGACGAGAATAAAC | 126 | This study |
| *tarL*-R | GCACCAATACCGAACAGAT |  |  |
| *16S rRNA*-F | CGGTGAATACGTTCYCGG | 128 | This study |
| *16S rRNA*-R | GGWTACCTTGTTACGACTT |  |  |

**Table S2. Primers for RT-PCR**
