# Supplementary figures and images for: Subinhibitory antibiotic concentrations promote the horizontal transfer of plasmid-borne resistance genes from Klebsiellae pneumoniae to Escherichia coli
Source: Front Microbiol. 2022 Nov 7;13:1017092. doi: 10.3389/fmicb.2022.1017092 (PMC9678054; doi:10.3389/fmicb.2022.1017092)

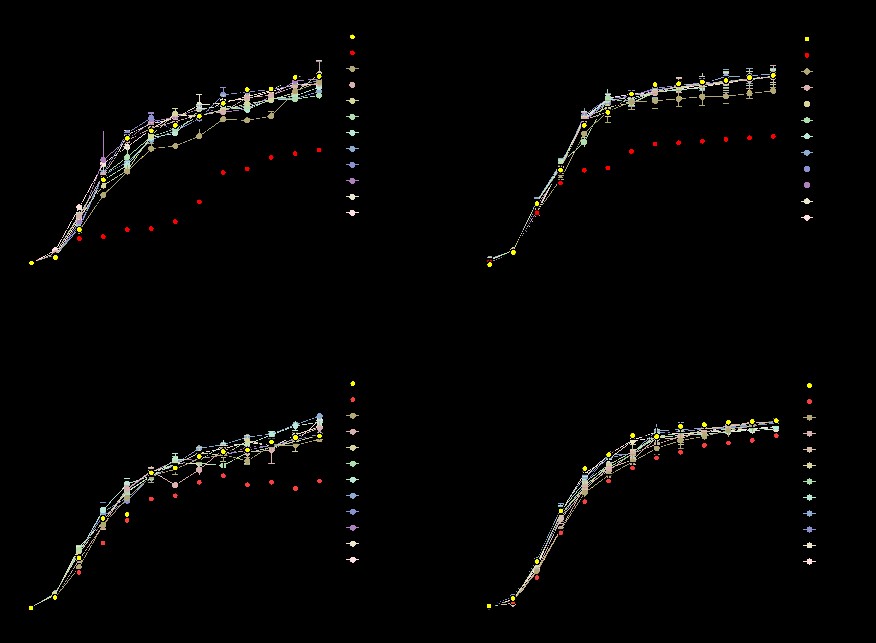

Supplement: Supplementary file 3 [file Image_1.jpg]

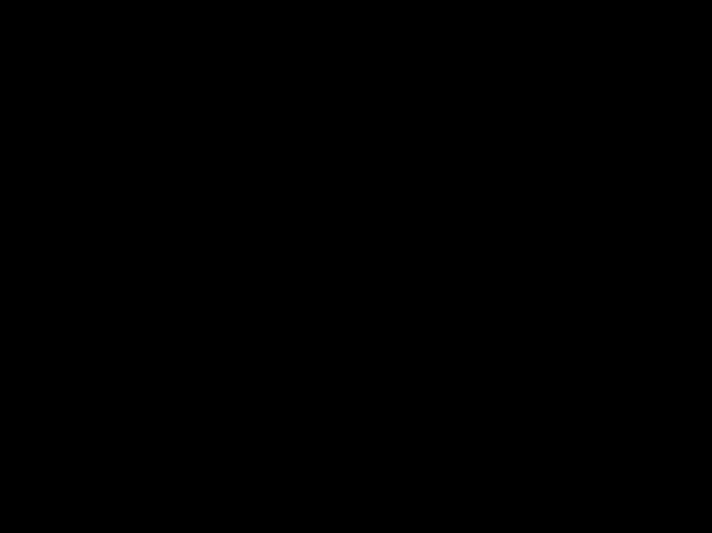

Supplement: Supplementary file 4 [file Image_2.jpg]

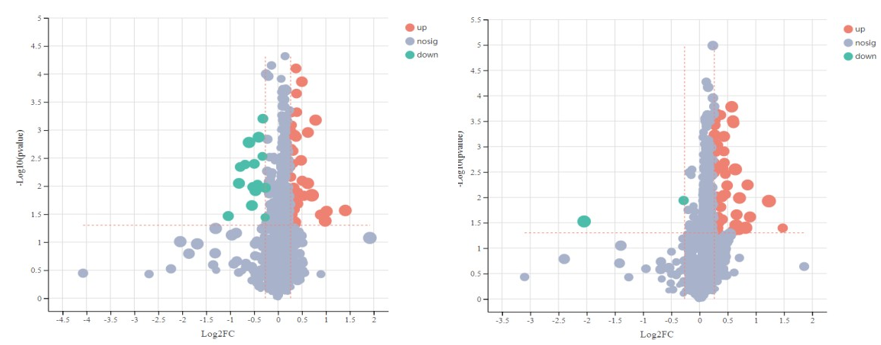

Supplement: Supplementary file 5 [file Image_3.png]
